# Specific Binding Peptides from Rv3632: A Strategy for Blocking* Mycobacterium tuberculosis* Entry to Target Cells?

**DOI:** 10.1155/2019/8680935

**Published:** 2019-04-14

**Authors:** Christian David Sánchez-Barinas, Marisol Ocampo, Luisa Tabares, Maritza Bermúdez, Manuel Alfonso Patarroyo, Manuel Elkin Patarroyo

**Affiliations:** ^1^Fundación Instituto de Inmunología de Colombia (FIDIC), Carrera 50 No. 26–20, 111321 Bogotá, Colombia; ^2^Universidad del Rosario, Carrera 24 No. 63C-69, 111321 Bogotá, Colombia; ^3^Universidad Nacional de Colombia, Carrera 45 No. 26-85, 11001 Bogotá, Colombia

## Abstract

Tuberculosis is an infectious disease caused by* Mycobacterium tuberculosis* (*Mtb*, i.e., the aetiological agent); the WHO has established this disease as high priority due to its ensuing mortality.* Mtb* uses a range of mechanisms for preventing its elimination by an infected host; new, viable alternatives for blocking the host-pathogen interaction are thus sought constantly. This article updates our laboratory's systematic search for antigens using bioinformatics tools to clarify the* Mtb* H37Rv Rv3632 protein's topology and location. This article reports a C-terminal region consisting of peptides 39255 and 39256 (^81^Thr-Arg^114^) having high specific binding regarding two infection-related cell lines (A549 and U937); they inhibited mycobacterial entry to U937 cells in a concentration-dependent manner. Rv3632 forms part of the mycobacterial cell envelope, formed by six linear synthetic peptides. Circular dichroism enabled determining the protein's secondary structure. It was also found that peptide 39254 (^61^Gly-Thr^83^) was a HABP for alveolar epithelial cells and inhibited mycobacteria entry to these cells regardless of concentration. Sera from active or latent tuberculosis patients did not recognise HABPs 39254 and 39256. These sequences represent a promising approach aiming at their ongoing modification and for including them when designing a multi-epitope, anti-tuberculosis vaccine.

## 1. Introduction

Tuberculosis has had a high incidence regarding the world's population; it affected more than 10 million people in 2017 and was the second cause of death by an infectious agent [1]. The appearance of new strains reported as having multi-drug resistance to antibiotics has hampered effective medical treatment [2].

 The* Mycobacterium bovis* Bacillus Calmette-Guérin (BCG) vaccine is currently endorsed by the WHO as it grants partial protection against* Mycobacterium tuberculosis* (*Mtb*), the main cause of tuberculosis [1]. This vaccine is only applied as a single dose to neonates to protect them against meningeal and miliary tuberculosis; however, it does not prevent infection in adults or the development of pulmonary tuberculosis [3, 4]. Several vaccination strategies have been advanced and 12 vaccine candidates are currently being tested in clinical phases (only three in phase 3) [5, 6]; these have been unconvincing so far and have proved uncertain when a long-term protective immune response has been assessed. They have not surpassed the current vaccine's performance.

A logical and rational alternative has been described by our institute (FIDIC) in its search for candidate antigens for an effective anti-tuberculosis vaccine. Such search has been based on identifying pathogen cell surface protein-derived peptides which are able to specifically bind infection target cells (i.e., the so-called high activity binding peptides, HABPs); these peptides are then tested to evaluate their ability to inhibit* Mtb* H37Rv entry* in vitro*.

This methodology has led to peptides being proposed for a synthetic anti-tuberculosis vaccine derived from the 34 pertinent proteins analysed to date [7–12]. The sequences involved in pathogen-host interaction represent candidates for inclusion when designing a synthetic vaccine. The objective is to induce an immune response which is capable of blocking* Mtb* interaction with infection target cells. No tests have yet been carried out for determining the antigenicity of HABPs identified to date (i.e., as they are important sequences for infection, they may not constitute T- or B/epitopes). This is why they must be modified after their antigenic capability has been evaluated so that an immune response is produced, thereby enabling the original sequence to be recognised, thus blocking* Mtb* entry.

Such research has led to some Rv3632 peptides being identified as promising components of a multi-epitope vaccine directed against mycobacterial-cell host interaction. The need for covering the greatest amount of mycobacterial access routes to infection target cells and for producing an effective immune response was taken into account.

Rv3632 forms a structural part of the* Mtb* H37Rv cell wall. It has been identified by mass spectrometry in proteomic analysis in extracts obtained with Triton X-114 [13] and has been recognised by mass spectrometry in protein membrane fractions and complete mycobacterial cell lysate, but not in culture filtrate [14]. Transposon site hybridisation (TraSH) analysis has reported that the gene encoding this protein is neither essential for mycobacteria [15], nor essential for mycobacterial* in vitro* growth [16].

Rv3632 participation in arabinogalactan (AG) biosynthesis has been described; this is an important cell wall component which is related to* Mtb* pathogenicity. It has also been suggested that Rv3632 plays a role in directing and stabilising polyprenyl-phospho-N-acetylgalactosamine synthase (PpgS) in the plasma membrane leading to polysaccharide biosynthesis [17].

This study reports Rv3632 C-terminal region sequences specifically binding to infection target cells and inhibiting mycobacterial entry which might be included when designing a multi-epitope anti-tuberculosis vaccine.

## 2. Materials and Methods

### 2.1. Bioinformatics Analysis of the Rv3632 Protein

The Rv3632 protein sequence was analysed* in silico* due to the importance of studying cell envelope proteins which participate in* Mtb* interaction with a particular host [18]. BLASTp (https://blast.ncbi.nlm.nih.gov/Blast.cgi) was used for analysing TB complex strains' homology using the protein sequence obtained from TubercuList (http://tuberculist.epfl.ch) database, using CLUSTAL Omega (https://www.ebi.ac.uk/Tools/msa/clustalo/) for multiple sequence alignment for identifying Rv3632 conserved regions [19]. ProtParam https://web.expasy.org/protparam/ was used for characterising parameters such as molecular weight, isoelectric point, and GRAVY index [20].

Rv3632 location had to be ascertained, as our approach focuses on cell envelop proteins. PSORTb v3.0.2 http://www.psort.org/psortb/ [21] was used for predicting this (as its use has been validated on Mtb proteins) [22], along with TBpred (http://crdd.osdd.net/raghava/tbpred/), a server exclusively designed for mycobacteria [23]. The SecretomeP 2.0 http://www.cbs.dtu.dk/services/SecretomeP/ [24] server was used for analysing the secretion route after topological analysis concerning the protein of interest's presence on mycobacterium surface had been determined. TMHMM 2.0 http://www.cbs.dtu.dk/services/TMHMM/ [25] and Phobius http://phobius.sbc.su.se/ [26] were used for analysing transmembrane topology/helices. The GPS-Lipid http://lipid.biocuckoo.org/webserver.php [27] server was used for predicting Rv3632's secondary structure elements and Phyre2 (http://www.sbg.bio.ic.ac.uk/phyre2) [28] was used for predicting whether Rv3632 had potential post-translational lipid modification sites. PSIPRED (http://bioinf.cs.ucl.ac.uk/psipred/) [29] was used for modelling three-dimensional structure (validated by Swiss model http://swissmodel.expasy.org/workspace/ [30, 31]. The STRING biological database (version 10.5) (https://string-db.org/) [32] was used for verifying whether Rv3632 interacted with other proteins forming part of the* Mtb* H37Rv genome.

### 2.2. *rv3632* Gene Presence and Transcription

The presence and transcription of the* rv3632* gene were analysed in normal* Mtb* H37Rv (ATCC 27294),* Mtb* H37Ra (ATCC 25177),* M. bovis* (ATCC 19210),* M. bovis* BCG (ATCC 27291), and* M. smegmatis* (ATCC 19420) mycobacterial culture conditions, taking into account that gene expression in mycobacteria is influenced by culture conditions [33, 34]. Middlebrook 7H9 culture medium (Difco Laboratories, Detroit, MI, USA) for* M. bovis* and* M. bovis* BCG was enriched with sodium pyruvate and without glycerol, whilst the medium for the other strains (H37Rv, H37Ra, and* M. smegmatis*) was supplemented with 10% oleic acid-albumin-dextrose-catalase (OADC). The cultures were incubated at 37°C for 10-20 days; once this time had elapsed (i.e., exponential (log) growth phase), they were harvested (OD_600_ 0.5-1.0) by spinning at 13,000 g for 40 minutes and the pellet was then suspended in 1X PBS and stored at -20°C until use.

An Ultra Clean Microbial DNA isolation kit (MoBio Laboratories Inc., Carlsbad, CA, USA) was used for extracting bacterial genomic DNA (gDNA); its quality was verified by PCR using* hsp65* constitutive gene primers (forward 5'-ACCAACGATGGTGTGTCCAT-3' and reverse 5'-CTTGTCGAACCGCATACCCT-3') and forward 5'-GATCATCGGGTTGCTGTTCT-3' and reverse primers 5'-CGCATGTAGGTGCTCAGTGT-3' for corroborating* rv3632* gene transcription and presence.

The TRIZOL (Invitrogen) method was used for isolating RNA from the strains analysed in this research for evaluating* rv3632* gene transcription; this was then treated with DNase I (Invitrogen). SuperScript III Reverse Transcriptase (Invitrogen) was used for complementary DNA (cDNA) synthesis.

A negative synthesis control was included for each sample by replacing reverse transcriptase with diethyl pyrocarbonate (DEPC)-treated water. 1 *μ*L template cDNA was used for PCR amplification when using GoTaq DNA polymerase (Promega, Madison, USA), 1.5 mM MgCl_2_, 1 mM dNTPs, and 1 *μ*L of each primer. Thermocycling conditions were 95°C for 5 min, followed by 35 cycles at 58.4°C for 45 s, 72°C for 30 s, 95°C for 30 s, and a final extension step at 72°C for 10 min. The amplification products were run on a 2% agarose gel and SYBR Safe (Invitrogen, Carlsbad, CA) was used for visualising them; the images were recorded on a Bio-imaging Systems photo-documenter.

### 2.3. Rv3632 Protein Expression in* Mycobacterium tuberculosis* H37Rv

BepiPred 1.0 server software (http://www.cbs.dtu.dk/services/BepiPred-1.0/) [35] was used for analysing the protein's sequence. This led to identifying B-epitope regions for obtaining the polyclonal antibodies needed for determining the protein's location (i.e., after having been inoculated into a murine model). Three inoculations with the polymeric peptide identified as B-cell epitope (at 20-day intervals) were thus made in female BALB/c mice at 80 *μ*g final concentration and mixed in a 1:1 ratio with incomplete Freud's adjuvant (Sigma, St. Louis, USA) to verify Rv3632 expression in the* Mtb* H37Rv strain in normal culture conditions. This polymeric peptide was obtained by oxidation to form a disulfide bond with the terminal residues of cysteine containing the peptide; its complete polymerisation was confirmed by Ellman's test (5,5-dithio-bis- [2-nitrobenzoic acid]) at pH 8.0.

Immunochemical tests using the polyclonal sera enabled determining Rv3632 expression and possible localisation in* Mtb* H37Rv. Western blot involved sonicating the mycobacteria for 15 minutes at 80% amplitude, twice, in lysis buffer (0.69 *μ*g/mL pepstatin, 0.5 *μ*g/mL leupeptin, 0.2 nM PMSF, 1 mg/mL DNase, 1 mg/mL RNase, and 10 mg/mL lysozyme in 1X PBS). This was followed by spinning at 13,000 rpm for 30 minutes at 4°C. The supernatant was recovered and inactivated by boiling for 90 minutes. Complete lysate proteins were separated by electrophoresis on 12% acrylamide denaturing gel and transferred to a 0.45 *μ*m nitrocellulose membrane (BIO-RAD, USA). They were then incubated for 2 hours at room temperature (RT) with polyclonal serum diluted 1:20, washed five times with 1% TBS-Tween 20 at pH 7.4, and then incubated for 1 hour with anti-mouse IgG secondary antibody-alkaline phosphatase (Vector Laboratories) in 1:10,000 dilution to be revealed using the BCIP/NBT system (5-bromo-4-chloro-3-indolyl-phosphate/nitro blue tetrazolium) (Promega, Madison, USA).

A HITACHI HU-12A transmission electron microscope was used for the immunoelectron microscopy (IEM) assay.* Mtb* H37Rv were fixed using a 4% paraformaldehyde/0.5% glutaraldehyde solution for two hours at 4°C. The pellet was then dehydrated in ethanol in a 50%-100% ratio and embedded in LR-white hard-grade resin (Sigma). This was polymerised using a specific cold catalyst [36]. A microtome was then used for cutting the resin block into 400 nm sections which were then placed on nickel grids. The grids were blocked with 5% SFB and 0.1% Tween 20 for 30 minutes, followed by immersion in a 1:20 primary antibody solution at 4°C/ON. The grids were washed several times with 0.5% BSA and 0.1% Tween 20 solutions and then incubated with anti-mouse IgG antibody labelled with 5nm colloidal gold (Sigma) for 1 hour at RT in 1:400 dilution [7]. The samples were incubated in 12% uranyl acetate for 15 minutes in the dark before being analysed by microscope.

### 2.4. Rv3623 Peptide Secondary Structure Determination

The Rv3632 protein is formed by six sequential, non-overlapping 20-residue-long peptides (coded 39251 to 39256); these were obtained by solid phase multiple synthesis, purified by reverse-phase, high performance liquid chromatography (RP-HPLC), and characterised by MALDI-TOF mass spectrometry using *α*-Cyano-4-hydroxycinnamic acid (*α*-CCA) as matrix [37]. ^113^Cys was replaced by Thr to avoid polymerisation, oxidation, or cyclisation reactions during peptide synthesis. The peptides were dissolved at 5 x 10^−6^ M concentration in 30% (v/v) trifluoroethanol for ascertaining their secondary structure; they were read in triplicate on a JASCO J-810 spectropolarimeter, giving ellipticity (*θ*) values measured in millidegrees (mdeg) for a spectral scan ranging from 190 to 260 nm. SELCON3, CDSSTR, and CONTINLL [38] were then used for deconvoluting the data for estimating each peptide's secondary structure elements, expressed as *α*-helices, folded *β*-sheets, and random coils.

### 2.5. Receptor-Ligand Assay 

Rv3632 peptides' capability for binding to* Mtb* infection target cells (A549 and U937) was estimated following already described methodology [7]. The peptides were radiolabelled by adding a Tyr residue to those sequences which did not contain it, labelling them with 5 *μ*L Na^125^I radionuclide, having 100 mCi/mL activity, and then separating the reaction products on a Sephadex G10 column. Briefly, in receptor-ligand binding assays, 1.2 *∗* 10^6^ cell/well were incubated for 2 hours (37°C for A549 and 4°C for U937 cell lines), with increasing concentrations of radiolabelled peptide (0-950 nM) in the presence or absence of excess unlabelled peptide (30 *μ*M). After incubation, cells were separated by spinning at 7,000 rpm for 5 minutes on dioctyl phthalate (DOTP)/dibutyl phthalate (DBP) (60:40, density 1.015 g/mL). The supernatant was discarded and pellet radioactivity was measured by gamma counter (Packard Cobra II Gamma Counter, Packard Instrument Co., Meriden, CT, USA).

Radiolabelled peptide bound to cells in the absence of non-radiolabelled peptide was called total binding and nonspecific or inhibited binding referred to bound radiolabelled peptide in the presence of excess (1 mg/mL) non-radiolabelled peptide; the difference between total binding and nonspecific binding gave specific binding. A peptide was considered to have high specific binding activity (HABP) when the slope of the specific binding line was greater than or equal to 1%. Saturation tests were used for obtaining HABP-cell interaction physicochemical constants (increasing the range of added radiolabelled peptide 0-8, 000 nM), following the methodology previously described for protein peptide screening. HABP dissociation constants (K_D_), Hill coefficients (n_H_), and cell binding sites were determined. Each assay was done in triplicate.

### 2.6. Inhibiting* Mycobacterium tuberculosis* Entry to Target Cells

Tuberculosis infection target cell lines (A549 and U937) were incubated (2.5 *∗* 10^5^ cell/well) at 37°C with 5% CO_2_ until monolayer formation; the medium was removed and placed in contact with Rv3632 peptides which had previously been determined as HABPs, involving increasing concentrations (2, 20 and 200 *μ*M) over a 2-hour period. The alveolar epithelial cells (A549) were incubated in standard conditions [7, 39], but the macrophages (U937) were maintained at 4°C to reduce their phagocytic activity. The cells were then infected with* Mtb* H37Rv at 1:75 multiplicity of infection (MOI); the cells were left in contact with the bacteria overnight in standard culture conditions. The medium was removed from the wells on the following day and the monolayers were washed with 1 X PBS at pH 7.3; adhered cells were removed with trypsin and an aliquot was plated in Middlebrook 7H10 medium with OADC and incubated at 37°C. Colony forming units (CFU) were counted 20 days later. Untreated cells were used as infection control (taking 100% infection) and 30 *μ*M cytochalasin as inhibition control; each assay was done in triplicate.

Rv3632 peptides' cytotoxic effect was evaluated by viability assay involving MTT (3- (4,5-dimethylthiazol-2-yl) -2,5-diphenyltetrazolium bromide) tetrazolium reduction, where 5 x 10^4^ cells/well were incubated for 2 hours with each peptide at 20 and 200 *μ*M in 1X PBS, using the previously mentioned incubation conditions for the invasion assay; 10% SDS was used as cytotoxicity control. After incubation time had elapsed, 10*μ*L MTT were added to each well and left for 12 hours; the formazan crystals so formed were dissolved with sodium dodecyl sulfate (SDS) for reading absorbance at 570nm on a spectrophotometer (Multiskan GO, Thermo Scientific). Multiskan GO (v. 3.2) software was used for analysing the data.

### 2.7. Evaluating Rv3632 Synthetic Peptides' Antigenicity

An enzyme-linked immunosorbent assay (ELISA) was used for evaluating the Rv3632 peptide's antigenic capability, using 10 sera from asymptomatic latent TB (LTB) patients having had a positive QuantiFERON test result, 5 sera from patients having active tuberculosis (ATB, based on conventional microbiological diagnosis), and 10 sera from healthy donors (HD). Peptides were immobilised in triplicate on a polystyrene 96-well tissue culture plate and incubated for 12 h at 4°C. After three washes with PBS-Tween and water, nonspecific sites were blocked with 5% skimmed milk in PBS-Tween for 2h; 3 washes were made with 0.05% PBS-Tween and water. The patients' plasma was placed in 1:100 dilution for 2 hours at 37°C. Anti-human IgG secondary antibody (MP Biomedicals) was then added at 1:5,000 dilution and incubated at 37°C for 1 h before being revealed by TBM-peroxidase system to be read at 450 nm.

## 3. Results

### 3.1. Bioinformatics Analysis

**Figure 1:**
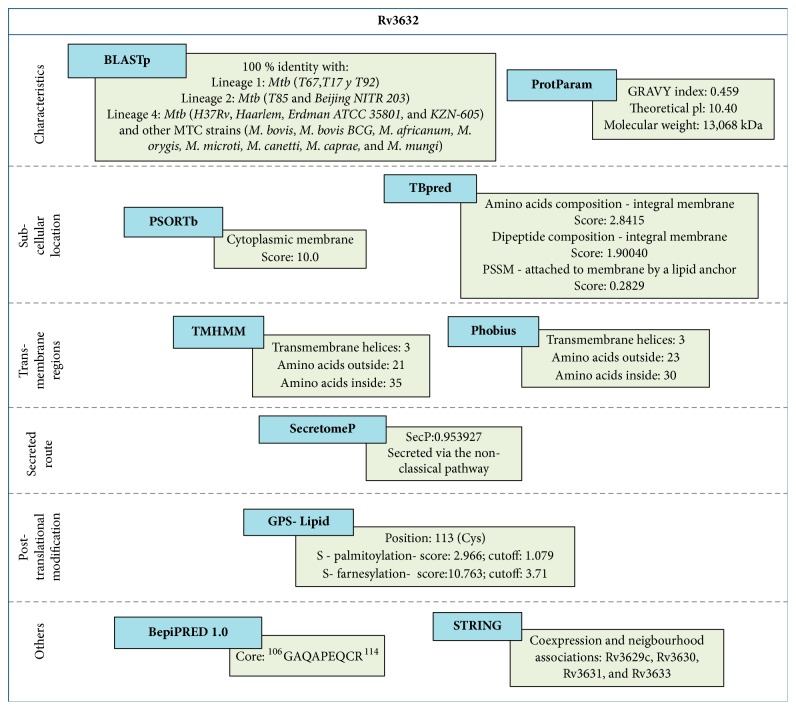
Rv3632 protein's characteristics, showing the protein's predicted localisation, topology and other characteristics.

Analysing Rv3632 amino acid (aa) sequence multiple homology revealed 100% identity with* Mtb* complex (MTC) strains:* M. bovis*,* M. bovis BCG*,* M. africanum*,* M. orygis*,* M. microti*,* M. canetti*,* M. caprae*, and* M. mungi* (Figure 1). Identity was also shown with clinical isolates from three* Mtb* lineages:* Indo-Oceanic* lineage (*T67*,* T17*, and* T92*),* East Asian* lineage (*T85* and* Beijing NITR203*), and* Euro-American/African* lineage (*H37Rv*,* Haarlem*,* Erdman ATCC 35801*, and* KZN-605*) [40].

The ProtParam platform was used for computing the Rv3632 sequence, giving a 13,068 kDa molecular weight, 10.40 isoelectric point, and 0.459 grand average of hydropathicity (GRAVY), or hydrophobicity index, indicating that the protein had little association with the membrane, as being characteristic of hydrophobic proteins [20]. Nevertheless, the PSORTb subcellular localisation prediction tool predicted that Rv3632 formed part of the mycobacterium's cytoplasmic membrane. TBpred predicted that Rv3632 would be an integral membrane protein (IMP) by analysing aa composition (score: 2.84) and dipeptide composition (score: 1.90). It also seemed to be a lipid-anchored protein as a position-specific scoring matrix (PSSM) gave a score of 0.28 (Figure 1).

TMHMM 2.0 software was used for predicting transmembrane topology/helices; it predicted three transmembrane helices for Rv3632 between positions ^4^Ile-Arg^21^, ^30^Ala-Leu^48^, and ^68^Leu-Tyr^85^, confirmed by the Phobius web server, but in positions ^6^Val-Arg^23^, ^30^Ala-Leu^48^, and ^68^Leu-Phe^88^. The difference in clarifying transmembrane regions depends on the algorithm and each predictor's parameters (Figure 1). It was found that Rv3632 did not have a proteolytic cleavage signal peptide to be transported to the membrane by classical route; however, the SecretomeP v2.0 server predicted that the protein would be secreted via non-classical pathway (>0.5 threshold value) (Figure 1). Mycobacterial surface proteins can be modified by glycosylation or the addition of lipophilic groups; the GPS-Lipid predictor suggested two possible lipid modifications for the Rv3632 sequence: S-palmitoylation (score: 2.97) and S-farnesylation (score: 10.76), both located in ^113^Cys. It did not predict O-glycosidic modifications (Figure 1).

The STRING database (v10.5) predicted functional associations for Rv3632 with* Mtb* H37Rv proteins, such as Rv3629c, Rv3630, Rv3631, and Rv3633, regarding both proximity and co-expressed and simultaneous associations (Figure 1) [32].

### 3.2. The* rv3632* Gene Was Present and Transcribed in* Mtb* Strains

**Figure 2:**
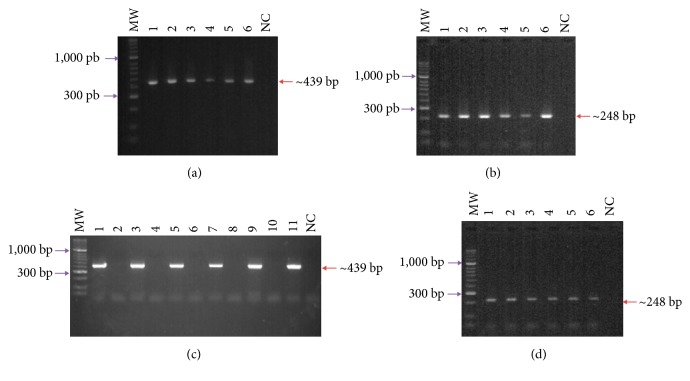
*rv3632* gene presence and transcription. (a) Amplification of* hsp65* gene from gDNA isolated from the following: (1)* M. tuberculosis* H37Rv; (2)* M. tuberculosis* H37Ra; (3)* M. bovis*; (4)* M. bovis* BCG; (5)* M. smegmatis*; (6) PC: positive PCR control; NC: negative PCR control. MWM: 50 bp molecular weight marker (b) Amplification of* rv3632* gene from gDNA isolated from the following: (1)* M. tuberculosis* H37Rv; (2)* M. tuberculosis* H37Ra; (3)* M. bovis*; (4)* M. bovis* BCG; (5)* M. smegmatis*; (6) PC: positive PCR control; NC: negative PCR control. (c)* hsp65* gene amplification of cDNA from the following: (1)* M. tuberculosis* H37Rv plus synthesis; (2)* M. tuberculosis* H37Rv minus synthesis; (3)* M. tuberculosis* H37Ra plus synthesis; (4)* M. tuberculosis* H37Ra minus synthesis; (5)* M. bovis* plus synthesis; (6)* M. bovis* minus synthesis; (7)* M. bovis* BCG plus synthesis; (8)* M. bovis* BCG minus synthesis; (9)* M. smegmatis* plus synthesis; (10)* M. smegmatis* minus synthesis; (11) PC: positive PCR control (*M. tuberculosis* H37Rv gDNA); NC: negative PCR control; MWM: 50 bp molecular weight marker. (d)* rv3632* gene amplification of cDNA from the following: (1)* M. tuberculosis* H37Rv; (2)* M. tuberculosis* H37Ra; (3)* M. bovis*; (4)* M. bovis* BCG; (5)* M. smegmatis*; (6) PC: positive PCR control; NC: negative PCR control. MWM: 50 bp molecular weight marker.

Good integrity was shown regarding samples of gDNA from the mycobacteria used in this study, as corroborated by constitutive* hsp65* gene expression of around 439 bp (Figure 2(a)).  Figure 2(b) shows a 248 bp amplification product, showing the* rv3632* gene in* Mtb* H37Rv,* Mtb* H37Ra,* M. bovis*, and* M. bovis* BCG strains belonging to the MTC and* M. smegmatis* which is a non-pathogenic strain. The constitutive* hsp65* gene was also used as transcription control for each mycobacterial strain; it was confirmed that there had been no contamination due to gDNA (Figure 2(c)). The 248 bp amplification fragment was observed in* Mtb* H37Rv,* Mtb* H37Ra,* M. bovis*, and* M. bovis* BCG strains; a lower intensity signal was observed for the* M. smegmatis* strain. The assay indicated that the gene was transcribed in the normal mycobacterial culture conditions analysed here (Figure 2(d)).

### 3.3. The Rv3632 Protein Was Expressed on* Mtb* Cell Envelope

**Figure 3:**
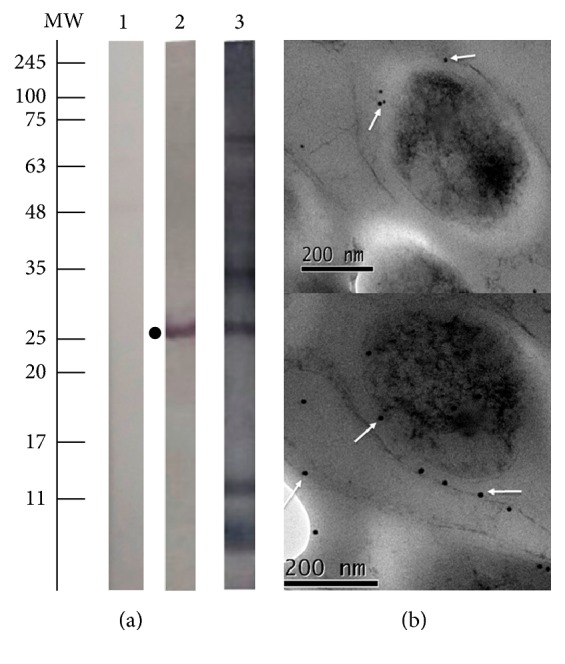
Detecting Rv3632 on mycobacterial surface. (a) Western blot recognition of Rv3632 in* M. tuberculosis* H37Rv. Lane 1: pre-immune sera. Lane 2: post-third immunisation of polymer 40057 against* Mtb* H37Rv lysate. Lane 3: recognition of hyper-immune control sera (obtained by inoculating* Mtb* H37Rv sonicate). Complete* Mtb* H37Rv protein lysate was used as antigen. (b) IEM assay localisation of Rv3632 on* Mtb* H37Rv envelop. Electron density was observed on bacillus surface due to colloidal gold-labelled antibody, indicated by white arrows.

The polyclonal sera obtained from inoculating polymer 40057 (CG-^96^ARIARALALEGAQAPEQTR^114^-GC), identified as B-cell epitope (Figure 1), led to recognition of a single band around 25 kDa (Figure 3(a), lane 2), as being almost twice the theoretical weight for Rv3632 (13 kDa) in* Mtb* H37Rv lysate, whilst no recognition was observed for pre-immune sera (Figure 3(a), lane 1). IEM confirmed Rv3632 presence on mycobacterial envelope (Figure 3(b)); electron-dense particles were observed (i.e., recognition of colloidal gold-labelled anti-mouse IgG antibody).

### 3.4. Rv3632 Protein Peptides Having High Specific Binding (HABPs) Bound to A549 and U937 Target Cells

**Figure 4:**
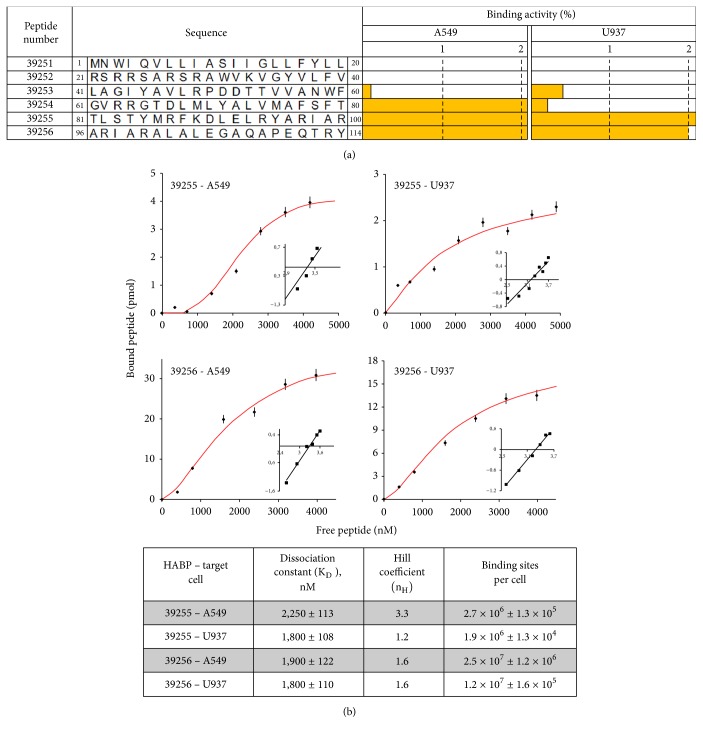
Rv3632 peptides' specific binding to infection target cells. (a) Rv3632 synthetic peptides had specific binding to A549 and U937cells. The bars show the percentage of specific binding to target cells; they were considered HABPs if this was ≥ 1%. (b) HABPs 39255 and 39256 saturation curves for the cell lines used here; the physicochemical constant values for this interaction are shown on the right-hand side.

The Rv3632 protein sequence consisted of 114 aa; six 20-aa-long peptide sequences were obtained from them for identifying these fragments' interaction with A549 epithelial cell (ATCC CLL-185) and U937 monocyte-derived macrophage (ATCC CRL-2367) receptors.  Figure 4(a) shows the specific binding results for each synthetic peptide regarding the cell lines used here. Peptides having ≥1% specific binding activity were considered target cell HABPs. HABPs for both cell lines comprised the protein's C-terminal region between aa 81Thr and Arg114, whilst peptide 39254 (^61^GVRRGTDLMLYALVMAFSFT^80^) was only a HABP for the A549 alveolar epithelial cell line.

Each peptide's saturation curves enabled determining the physicochemical constants regarding HABP-receptor cell interaction for the infection target lines. HABP dissociation constants (K_D_) were in the 1,800-2,250 nM range and Hill coefficients (n_H_) for these interactions were >1, indicating positive cooperativity for HABP binding. Between 1.9 x 10^6^ and 2.7 x 10^6^ receptor sites were calculated per cell for peptide 39255 and around 10 times more sites for HABP 39256 binding. Future studies should be aiming at determining the nature of HABP receptor sites ( Figure 4(b)).

### 3.5. Rv3632 Peptide Secondary Structure

**Figure 5:**
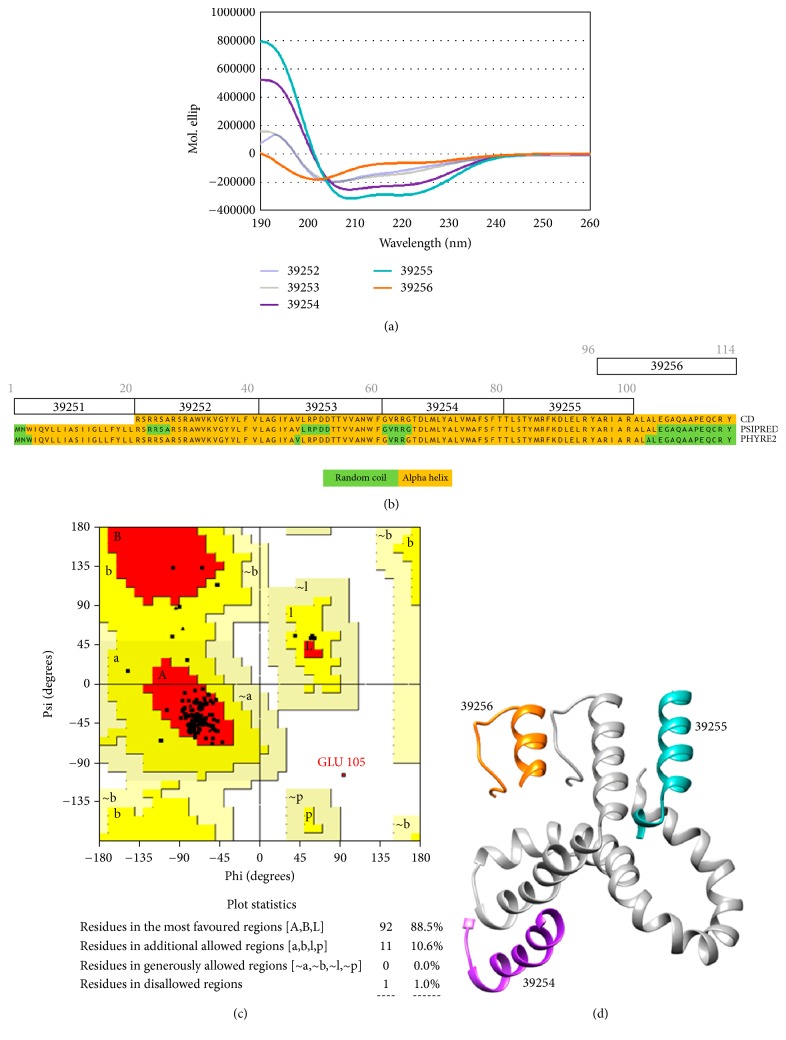
Rv3632 protein structural analysis: (a) Rv3632 peptides circular dichroism. (b) PSIPRED predicted Rv3632 structure secondary. (c) Evaluating the Phyre2 structural model by Ramachandran plot; red areas indicate favoured regions and those in yellow permitted regions. (d) Phyre2 prediction of Rv3632's 114 aa three-dimensional structure (Chimera 1.11.2-model server prediction model validated by Swiss model). HABPs are highlighted in the figure.

Circular dichroism [38, 41] was used for experimentally defining Rv3632 peptides' secondary structure; it was seen that *α*-helix structural elements predominated in all peptides, characterised by having a 192 nm maximum molar ellipticity and two minimums at 209 nm and 222 nm. Peptide 39253 (^41^LAGIYAVLRPDDTTVVANWF^60^) secondary structure had 2% folded *β*-sheets and peptide 39255 (^81^TLSTYMRFKDLELRYARIAR^100^) 1.5% (Figure 5(a)). Such data was consistent with the prediction obtained with PSIPRED and Phyre2 predictions, demonstrating that the peptides had a strong tendency towards *α*-helix structure, accompanied by short random coil regions (Figure 5(b)).

Phyre2 modelling of protein 3D structure and Swiss model validation giving a 0.121 QMEAN score led to a reliable structure, 88.5% of the residues being located in the Ramachandran plot's most favoured regions (Figure 5(c)). Regarding aa, 10.6% were located in allowed regions, resulting in an energetically stable structure. HABP location regarding the structure predicted by Phyre2 for Rv3632 revealed peptides 39254, 39255, and 39256 exposure, thereby enabling their interaction with the cell lines used here (Figure 5(d)). Regarding each peptide's experimentally determined structural elements, similarity was found with the Phyre2's predicted three-dimensional structure for Rv3632; no difference was found concerning that predicted for secondary structure.

### 3.6. Rv3632 HABPs Blocked* Mtb* Entry to A549 Epithelial and U937 Macrophage Target Cells

**Figure 6:**
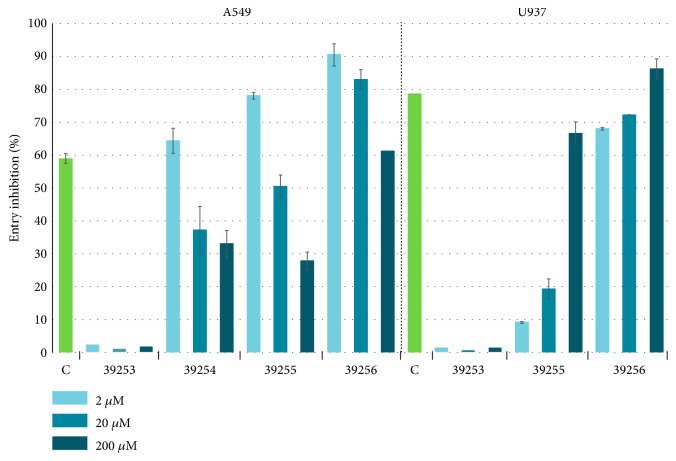
Rv3632 HABP-mediated inhibition of* Mtb* H37Rv entry to target cells. Percentage inhibition of* Mycobacterium tuberculosis* H37Rv entry to infection target cells by HABPs (at increasing concentrations: 2, 20, and 200 *µ*M). Cytochalasin D was used as positive inhibition control at 30 *µ*M (C). Peptide 39253 was used as non-HABP control for both cell lines used in this study.

HABPs 39254, 39255, and 39256 (at 2, 20, and 200 *μ*M concentrations) were brought into contact with epithelial and (A549) macrophage cell lines (U937) to evaluate their capability for specifically inhibiting* Mtb* H37Rv entry. Cytochalasin D was used as inhibition control and peptide 39253 (^41^LAGIYAVLRPDDTTVVANWF^60^) which had no binding as negative control. Cytochalasin inhibited mycobacterial entry to A549 epithelial cells by 60% and U937 monocyte-derived macrophages by 80%. HABPs 39255 and 39256 inhibited mycobacteria entry to U937 macrophages by up to 80% in a concentration-dependent manner and inhibited entry to A549 cells inversely to peptide concentration. Alveolar epithelial HABP 39254 inhibited mycobacterial entry inversely to peptide concentration (Figure 6). Peptide 39253 used as control did not bind to the cell lines and was not involved in* Mtb* H37Rv entry to infection target cells.

**Figure 7:**
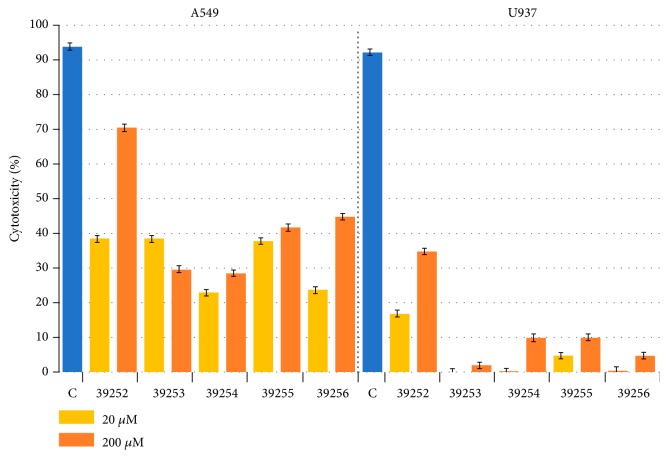
Rv3632 peptides' cytotoxicity. The percentage of each peptide's cytotoxicity for A549 and U937cells, evaluated at 20 *µ*M y 200 *µ*M concentrations. SDS was used as cytotoxicity control (C).

Peptide 39252 reached 70% cytotoxicity against the A549 alveolar epithelial cell line at 200*μ*M in cytotoxicity assays, whilst the other Rv3632 peptides had around 40% cytotoxic effect on the same cells. There was no significant cytotoxic peptide effect on the U937 macrophage line, except for peptide 39252 (Figure 7).

### 3.7. Evaluating Rv3632 Synthetic Peptides' Antigenicity

**Figure 8:**
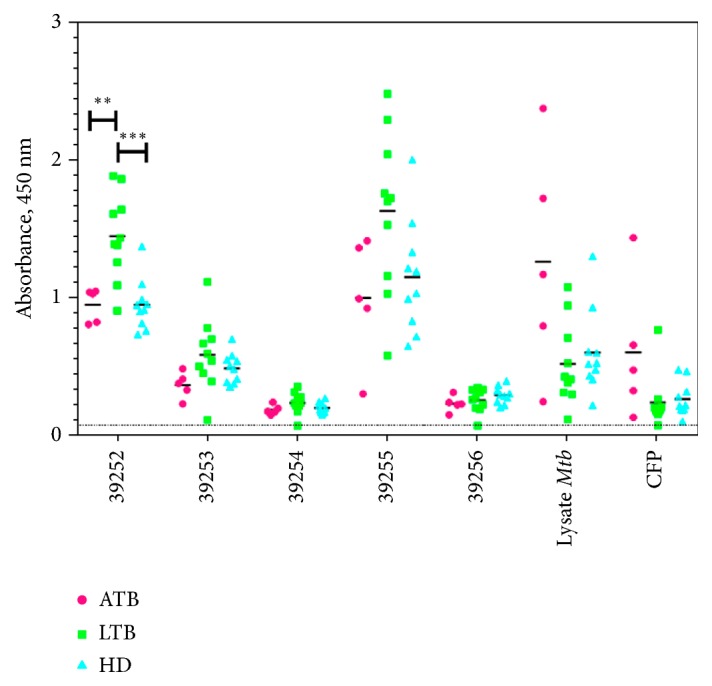
Antigenic recognition of Rv3632 peptides. Evaluating recognition of sera from individuals suffering from active tuberculosis (ATB), having latent tuberculosis (LTB), and healthy donors (HD).* Mtb* H37Rv lysate and proteins released by mycobacteria in Sauton's medium (CFP) were also used as antigens. Absorbances were above the threshold (0.0503) calculated in the absence of antigen (dotted line below the X axis). ^*∗∗*^*p* > 0.028; ^*∗∗∗*^*p* > 0.0003 Tukey's multiple comparison test. GraphPad Prism v.7.0. was used to draw the figure.

In our experience (malaria), HABPs neither are recognised by infected individuals' sera nor do they induce an immune response. Their sequences must thus be modified to make them immunogenic and antigenic, thereby triggering a protective immune response [42, 43]. It seems that a similar phenomenon regarding “immunological silence” may occur in tuberculosis where sequences of importance regarding the host-pathogen interaction are not immunologically relevant. It was thus necessary to evaluate whether Rv3632 peptides were antigenic when designing a multi-epitope vaccine against TB. Donors' sera were therefore used for determining recognition of the sequences; they were classified as having active tuberculosis (ATB), being in latent stage (LTB) or being healthy donors (HD).  Figure 8 shows that N-terminal region peptide 39252 (^21^RSRRSARSRAWVKVGYVLFV^40^) (not considered as a HABP) was recognised by LTB donor sera and had a significant difference regarding recognition of HD sera and ATB patients' sera. HABP 39255 (^81^TLSTYMRFKDLELRYARIAR^100^) was recognised by antibodies from LTB and ATB patients and HD, no significant differences being observed between the populations studied. HABPs 39254 and 39256 were not recognised by the sera being used, which might have been associated with* Mtb* H37Rv binding and entry to cell lines.* Mtb* H37Rv lysate was recognised by ATB patients' sera, whilst the culture supernatant was not recognised by the sera being used.

## 4. Discussion

Tuberculosis continues to be one of the diseases having the highest impact on public health worldwide and the BCG vaccine has proved ineffective in protecting adults against pulmonary tuberculosis. As the amount of patients diagnosed with the tubercle bacillus continues to grow, the search for fresh vaccine alternatives continues to be an urgent item on health agendas globally. FIDIC's research represents a promising advance in designing a synthetic vaccine intended to control the pathogen-host interaction by blocking specific receptors by using synthetic peptides for a large range of aetiological agents.

This study contributes towards the search for protein sequences on* Mtb* bacterial envelope which are involved in the host-pathogen interaction and which can be included when designing a multi-epitope synthetic vaccine against TB. This study has thus proposed Rv3632-derived HABPs as candidate antigens for blocking specific interactions between target cell receptors and mycobacteria. Despite not having an established function to date, it was found that the* rv3632* gene is present and is transcribed in TCM complex strains and in* Mtb* lineages (being conserved amongst them), thereby suggesting that Rv3632 could be involved in the aetiological agent's virulence.

TBpred is a tool which can be widely used for the functional prediction of mycobacterial proteins since it predicts four subcellular locations (cytoplasmic, integral membrane, secreted, and attached to the membrane by a lipid anchor) [23]. Rv3632 was predicted to be located on cell membrane, probably anchored to this section by a lipid which would be in agreement with that reported by the GPS-Lipid server, C-terminal Cys113 being a palmitic acid coupling site or a prenylation. The sequence of the peptide used in obtaining the polyclonal serum is exclusive to Rv3632. The protein's location on the mycobacterial envelope (confirmed by electron microscopy) would have allowed it to come into contact with infection target cells; however, Western blot did not reveal a band close to the protein's theoretical molecular weight (13 kDa) which could have been related to the prediction of a modification having a hydrophobic group for its anchorage, in turn allowing it to be exported to the membrane. Western blot also revealed a band of around 25 kDa, suggesting that Rv3632 could be a protein dimer (i.e., part of a protein complex having higher molecular weight), taking the presence of ^113^Cys into account. Although STRING v10.5 predicted associations involving Rv3629c (39kDa), Rv3630 (43kDa), Rv3631 (26kDa), and Rv3633 (33kDa) being co-expressed and having a synergistic effect which has been described in arabinogalactan production by interaction with PPGs or Rv3631, such associations would not be responsible for the experimental results reported here.

A highly specific binding region consisting of the 55 aa between ^61^Gly and Tyr^114^ was identified when screening Rv3632 peptides' 114 aa in terms of interaction with infection target cells (i.e., being analogous to HABPs 39354, 39255, and 39256). When evaluating these HABPs' ability to block* Mtb* H37Rv entry it was clear that 39255 and 39256 inhibited* Mtb* entry to the U937 cell line in a concentration-dependent manner, having > 50% inhibition at 200 *μ*M concentration, without having a cytotoxic effect. Regarding A549 epithelial cells, HABPs 39254, 39255, and 39256 inhibited entry by ≥ 30% at 200 *μ*M regardless of concentration. Such pattern may be related to the peptide cytotoxic effect observed for this cell line; although cytotoxicity may influence entry inhibition data, the inhibitory activity of these HABPs is relevant to our approach. Peptide 39253 (considered as a control in this assay because it did not bind to target cells) did not influence* Mtb* entry to target cells, which was to be expected for non-binding sequences. Only HABPs characterised by inhibiting* Mtb* entry to infection target cells would be included when designing a synthetic peptide-based, multi-epitope anti-TB vaccine. Although receptor-ligand assays have highlighted positive cooperation and identified a large amount of binding sites, possible cell receptor nature and identity have not yet been clarified.

Studies have highlighted a humoral immune response in controlling* Mtb* infection during the disease's active phase [44, 45]; however, it has been demonstrated that sequences for specific binding to infection target cells which are conserved in* Mtb* strains have not been immunogenic. The ELISA assays used here determined that HABPs 39254 and 39256 (which inhibited* Mtb* entry* in vitro*) were not recognised by TB patients' antibodies and might not have been producing any type of protective response against* Mtb*. These two HABPs highlighted a previously described phenomenon known as conserved antigens' immunological silence [42].

Both of the bioinformatics approach and the experimental evidence were similar in terms of these binding sequences' secondary structure elements; alpha helices predominated throughout the entire protein and the high specific binding sequences were exposed. As our approach to the search for antigens which might be included when designing a synthetic anti-tuberculosis vaccine has been based on results obtained so far regarding the malaria model, it is proposed here that HABP structures must be modified by specifically replacing aa in the peptides' original sequences. This ensures a poly-proline-type structure having a better fit into the major histocompatibility (MHC) class II complex, thereby improving immunogenic and protective characteristics [43].

Healthy donors (HD) had antibodies against Rv3632 peptides; this may have resulted from cross-reactivity because they might have been in contact with environmental mycobacteria or the simple fact of HD having been vaccinated with BCG (i.e., as being the only vaccine validated and accepted by the WHO against* Mtb* and which is mandatory within Colombia's healthcare plan). Interestingly, there was differential recognition of peptide 39252 by HD individuals' sera, thereby enabling it to be differentiated from the sera of individuals classified as ATB and LTB. Its sequence can thus be considered when developing serodiagnostic tests for TB.

## 5. Conclusion

It can thus be concluded that Rv3632 was exposed on bacterial surface, taking into account that our interest lay in blocking the host-pathogen interaction; it is thus feasible that it may be involved in contact with* Mtb* infection target cells. Peptide 39256 (^96^TARIARALALEGAQAPEQCRY^114^) was identified as being relevant in interaction with target cells capable of inhibiting* Mtb* entry to target cells and specifically binding to them. This sequence was conserved and not recognised by antibodies, which is why it has been proposed that this peptide should be modified to increase its immunogenicity, thereby enabling it be recognised by the host's immune system to include it when designing a synthetic anti-tuberculosis vaccine.

## Data Availability

The data used to support this study's findings are available from the corresponding author upon request.
